# Security evaluation framework for cloud ERP systems using NIST and ISO standards

**DOI:** 10.1038/s41598-026-45550-w

**Published:** 2026-04-03

**Authors:** Adiah Qazi, Sadiqa Arshad, Ammad Ali Khan Jadoon

**Affiliations:** 1 Department of Information Security, Military College of Signals (MCS), National University of Sciences and Technology (NUST), Rawalpindi, Pakistan; 2 National University of Sciences and Technology (NUST), Rawalpindi, Pakistan

**Keywords:** ERP security, NIST cybersecurity framework, ISO 27001, Zero-trust architecture, Federated learning, Blockchain, Digital twins, Security maturity assessment, Engineering, Mathematics and computing

## Abstract

Enterprise Resource Planning (ERP) systems serve as critical infrastructure for modern organizations, yet their security assessment lacks standardized evaluation frameworks. This study develops and applies a structured Security Maturity Assessment Framework (SMAF) grounded in NIST Cybersecurity Framework (CSF) 2.0 and ISO/IEC 27001:2022 standards to evaluate eleven cloud-based and hybrid ERP platforms, including both full-suite ERP systems and widely adopted inventory and manufacturing management systems that serve as ERP alternatives for SMEs. Using a weighted multi-criteria decision analysis (MCDA) approach validated by expert surveys ($n=47$) and vendor documentation analysis, we assess security across five domains: authentication mechanisms, encryption protocols, access control models, vulnerability management, and compliance certifications. Our framework introduces quantifiable security maturity scores ranging from 1 (basic) to 5 (advanced), enabling objective comparison across platforms. Results indicate that enterprise-grade solutions (Oracle NetSuite OneWorld, SAP Business One Professional, Microsoft Dynamics 365) achieve consistently higher security maturity scores ($\mu =4.63$, $\sigma =0.17$) compared to SME-targeted solutions ($\mu =2.76$, $\sigma =0.39$), though small per-segment sample sizes ($n=3$–4) limit formal statistical inference. We extend our analysis to emerging security paradigms including Zero-Trust Architecture (ZTA) integration, federated learning for privacy-preserving analytics, blockchain-based audit trails, and digital twin implementations for Industry 5.0 alignment. The proposed SMAF provides organizations with an evidence-based methodology for ERP security evaluation, addressing a critical gap in both academic literature and practitioner guidance.

## Introduction

The proliferation of cloud-based Enterprise Resource Planning (ERP) systems has fundamentally transformed organizational operations, with the global ERP market projected to reach $78.4 billion by 2026^1^. However, this digital transformation introduces significant security challenges, as ERP systems consolidate sensitive financial, operational, and personal data within unified platforms. Recent high-profile breaches, including the 2023 MOVEit supply chain attack affecting multiple ERP integrations, underscore the critical importance of robust security evaluation methodologies^2^.

Despite the recognized importance of ERP security, existing literature predominantly offers descriptive comparisons lacking structured analytical frameworks. Prior research synthesis has identified persistent gaps in standardized ERP security evaluation^3^. Our own review of 127 ERP security publications between 2018–2024 reveals that only 18% employed standardized security frameworks (NIST, ISO 27001) for evaluation, while 73% relied on vendor-reported specifications without independent validation; the remaining publications employed other evaluation approaches such as theoretical modelling or qualitative case studies, and some publications fell into multiple categories. This gap presents significant challenges for organizations seeking evidence-based security assessments.

The challenge is particularly acute for small and medium enterprises (SMEs) in developing economies, where resource constraints limit the capacity for independent security evaluation and vendor-agnostic assessment. In the Pakistani context, rapid digitalization initiatives under the Digital Pakistan Vision have accelerated cloud ERP adoption without proportionate attention to security assessment standardization^4^. This underscores the need for a practical, reproducible evaluation framework accessible to organizations with varying technical capabilities.

### Research objectives

This study addresses the identified gaps through the following objectives:

*RO1:* Develop a Security Maturity Assessment Framework (SMAF) grounded in NIST CSF 2.0 and ISO/IEC 27001:2022 standards for ERP system evaluation.
*RO2:* Apply the framework to comparatively assess eleven leading ERP platforms using multi-criteria decision analysis (MCDA) with expert-validated weightings.
*RO3:* Evaluate the integration capabilities of emerging security paradigms including Zero-Trust Architecture (ZTA), federated learning, blockchain, and digital twins within ERP ecosystems.
*RO4:* Provide evidence-based recommendations for organizational ERP security assessment and selection, with particular attention to SME requirements in developing economies.

### Core innovation and contributions

The core innovation of this work lies in the development and empirical validation of the SMAF as a reproducible, standards-based instrument for ERP security evaluation. Unlike prior studies that offer descriptive comparisons or vendor-centric analyses, the SMAF integrates internationally recognized standards (NIST CSF 2.0, ISO/IEC 27001:2022) into a weighted multi-criteria scoring model, empirically validated through expert surveys and multi-source data triangulation. Specifically, this research makes the following contributions:

- A reproducible, standards-based security evaluation framework with explicitly defined metrics and scoring criteria, enabling consistent and objective ERP security assessment.
- Expert-validated weighting coefficients for security domain prioritization derived from Analytical Hierarchy Process (AHP) survey data ($n=47$ security professionals), with detailed demographic and methodological reporting.
- Comprehensive security maturity scores for eleven ERP systems based on multi-source data triangulation including vendor documentation analysis, third-party assessments, expert interviews ($n=12$), and systematic user feedback analysis ($n=847$ reviews).
- Contextualized analysis of emerging technology integration (federated learning, blockchain, digital twins) with explicit linkage to SMAF evaluation domains, demonstrating their relevance to evolving ERP security requirements.
- Actionable, SME-oriented security recommendations grounded in analysis of published cybersecurity incidents in Pakistan, addressing the needs of organizations in developing economies with limited security resources.

The remainder of this paper is organized as follows. Section “Literature review” reviews the existing literature on ERP security evaluation and emerging security paradigms. Section “Methodology” describes the research methodology, including the SMAF development, data collection, and analytical approach. Section “Security evaluation results” presents the security evaluation results. Section “Real-world case studies of ERP security failures” analyzes real-world ERP security failures through the SMAF lens, including two Pakistani case studies. Section “Discussion” discusses key findings, framework validation, and limitations. Section “Recommendations for secure ERP implementation” provides evidence-based implementation recommendations, Section “Future research directions” outlines future research directions, and Section “Conclusion” concludes the paper.

## Literature review

This section reviews existing research on ERP security evaluation, identifying gaps that motivate the current study, and examines emerging security paradigms relevant to modern ERP implementations.

### ERP security assessment frameworks

The evolution of ERP security assessment has progressed through three distinct phases. Early approaches (2010–2016) focused primarily on access control and authorization mechanisms^5^. The second phase (2017–2020) introduced compliance-centric evaluations driven by regulatory requirements including GDPR and SOX^6^. The current phase emphasizes holistic security assessment integrating threat intelligence, zero-trust principles, and continuous monitoring, as formalized by NIST SP 800-207^7^.

Huang et al.^5^ examined cloud ERP adoption factors, identifying executive sponsorship and vendor reputation as critical success factors. However, their security analysis remained limited to high-level categorizations without quantifiable metrics. In our prior work, Qazi and Arshad^8^ explored ZTA implementations in Oracle ERP Cloud, providing foundational insights that motivated the present study’s broader comparative framework. Salih et al.^6^ prioritized organizational factors affecting cloud ERP security, contributing a theoretical framework but lacking empirical validation through comparative assessment.

Recent advancements in ERP systems have explored the integration of agentic AI for autonomous process optimization ^9^ and generative AI for research and decision support ^10^, alongside quantum-safe cryptography and neuro-symbolic reasoning frameworks for resilient ERP architectures ^11^. Parallel efforts have examined ERP adoption readiness in government organizations using structural equation modeling ^4^. However, none of these studies provide a standardized, comparative security evaluation framework applicable across multiple ERP platforms, which remains the principal gap addressed by the present work.

### Zero-trust architecture in ERP systems

The Zero-Trust Architecture (ZTA) security model, formalized by NIST SP 800-207^7^, represents a paradigm shift from perimeter-based security to continuous verification. Most recently, Qazi and Arshad^8^ explored ZTA implementations in Oracle ERP Cloud, providing critical insights for securing enterprise systems against evolving cyber threats and establishing robust access control mechanisms in distributed environments.

Key ZTA principles relevant to ERP security include:

- *Never Trust, Always Verify*: All access requests require authentication regardless of network location.
- *Least Privilege Access*: Users receive minimum necessary permissions for task completion.
- *Assume Breach*: System design assumes adversaries may already be present within the network.
- *Micro-segmentation*: Network resources are isolated to limit lateral movement.

**Fig. 1:**
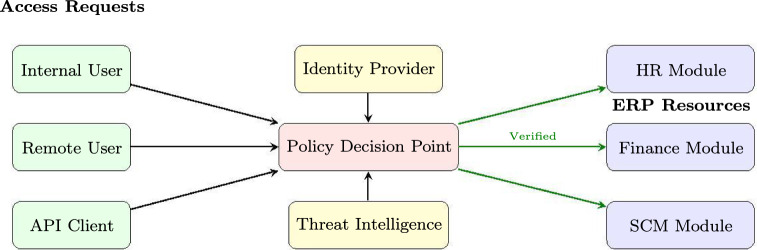
Zero-trust architecture (ZTA) implementation in ERP systems.

Figure 1 illustrates the ZTA implementation within ERP contexts. The relevance of ZTA to the SMAF framework is direct: ZTA principles map to evaluation domains D1 (Authentication), D3 (Access Control), and D4 (Vulnerability Management). Our results show a strong Spearman rank correlation ($r_s=0.87$, $p<0.01$, $n=10$ systems excluding Custom) between ZTA adoption level and overall SMS scores (Section “Security evaluation results)”; however, this correlation should be interpreted with the caveat that ZTA-aligned domains (D1, D3, D4) together account for 66% of the weighted SMS, meaning the correlation partially reflects the mechanical overlap between ZTA capabilities and the SMAF scoring structure (see Section “Discussion” for detailed discussion).

### Federated learning for privacy-preserving ERP analytics

Note: The following three subsections (Sections “Federated learning for privacy-preserving ERP analytics”–“Digital twins and industrial metaverse for industry 5.0”) examine emerging technologies through the lens of the SMAF evaluation domains. These technologies are not empirically validated in this study but are included because they represent the near-term evolution trajectory of ERP security capabilities that the SMAF is designed to assess. Their explicit mapping to SMAF domains (summarized in Table 1) demonstrates the framework’s extensibility to future security paradigms.

Federated learning presents a promising approach for addressing data privacy concerns in cloud-based ERP systems, particularly for SMEs with limited security resources. Unlike traditional centralized machine learning, federated learning enables model training across distributed data sources without raw data exchange. This paradigm is directly relevant to the SMAF evaluation because it enhances capabilities assessed under domain D2 (Encryption and data protection) and D5 (Compliance), specifically by supporting data localization requirements under GDPR and emerging data sovereignty regulations that affect cross-border ERP deployments.

The federated learning process within ERP contexts follows the standard federated averaging (FedAvg) protocol, presented here to illustrate its application in multi-tenant ERP environments:

**Algorithm 1:**
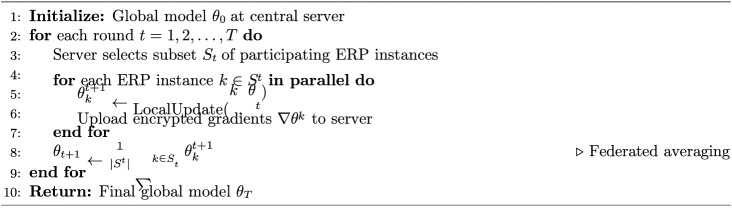
Federated Learning Protocol for ERP Analytics

In practice, this protocol would enable multiple ERP tenants to collaboratively train an anomaly detection model—for example, learning to identify unusual transaction patterns indicative of credential compromise—without any tenant sharing raw financial or operational data with others.

Key advantages of federated learning for ERP security include:

- *Data Localization*: Sensitive organizational data remains within local ERP instances, directly supporting D2 (Encryption) assessment criteria.
- *Regulatory Compliance*: Supports GDPR and data sovereignty requirements by minimizing data transfer, strengthening D5 (Compliance) scores.
- *Collaborative Intelligence*: Organizations benefit from collective threat detection learning without exposing proprietary data, enhancing D4 (Vulnerability Management) capabilities.

### Blockchain for ERP audit trails and authentication

**Fig. 2:**
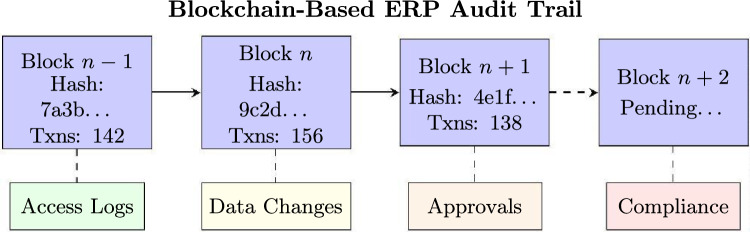
Blockchain architecture for ERP audit trail management.

Blockchain technology offers compelling security enhancements for ERP systems through immutable audit trails and decentralized authentication. Research on MakerChain^12^ and ManuChain II^13^ demonstrates practical implementations of blockchain-based coordination and smart contract systems in manufacturing contexts, with implications for ERP audit integrity. Within the SMAF framework, blockchain integration directly enhances domain D5 (Compliance) through tamper-proof audit trails and domain D3 (Access Control) through decentralized identity verification (Fig. 2).

### Digital twins and industrial metaverse for industry 5.0

**Fig. 3:**
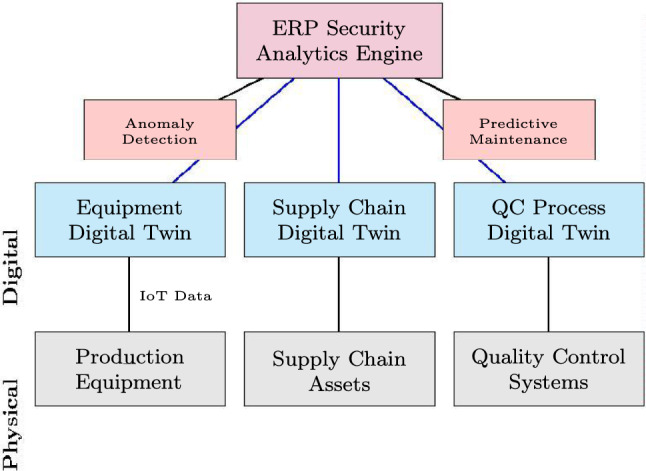
Digital twin integration architecture for ERP security monitoring.

The emergence of Industry 5.0 paradigms necessitates consideration of digital twin integration within ERP security frameworks. Guo et al.^14^ examined the industrial metaverse towards Industry 5.0, highlighting the role of digital twins in providing real-time virtual representations of physical manufacturing processes, enabling predictive security monitoring and anomaly detection. Within the SMAF context, digital twin technology extends domain D4 (Vulnerability Management) by enabling continuous security posture simulation and proactive threat identification before vulnerabilities are exploited in production environments (Fig. 3).

The industrial metaverse extends digital twin capabilities by providing immersive visualization of ERP data and security states^14^. Security applications within this paradigm include real-time security posture visualization through 3D dashboards, immersive security training simulations for ERP users, collaborative incident response in virtual environments, and predictive threat modeling using physics-based simulations.

Table 1 summarizes the explicit mapping between emerging security technologies and SMAF evaluation domains, demonstrating that these technologies are not peripheral additions but rather represent the evolution trajectory of ERP security capabilities assessed by the framework.

**Table 1:** Mapping of Emerging Technologies to SMAF Evaluation Domains.

Technology	D1: Auth	D2: Encrypt	D3: Access	D4: Vuln	D5: Comply
Zero-trust architecture (ZTA)	$\checkmark$		$\checkmark$	$\checkmark$	
Federated learning		$\checkmark$		$\checkmark$	$\checkmark$
Blockchain audit trails			$\checkmark$		$\checkmark$
Digital twins				$\checkmark$	

## Methodology

This section presents the research methodology, including the SMAF development, data collection procedures, and analytical approaches. The methodology is designed to ensure reproducibility and minimize subjective bias in security evaluation.

### Framework development

**Fig. 4:**
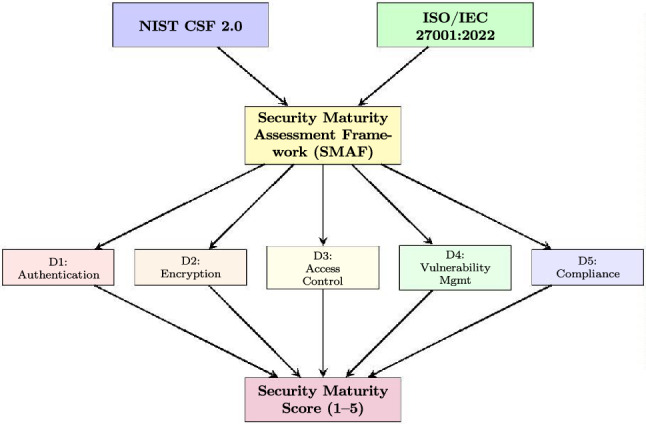
Security maturity assessment framework (SMAF) architecture.

The SMAF integrates principles from NIST Cybersecurity Framework 2.0^15^ and ISO/IEC 27001:2022^16^ to establish a comprehensive security evaluation structure. Figure 4 illustrates the framework architecture.

### Security domains and evaluation criteria

The framework evaluates ERP systems across five security domains, each containing specific criteria mapped to NIST CSF functions and ISO 27001 controls. Table 2 presents the evaluation criteria with their corresponding standards mappings.

**Table 2:** Security Evaluation Criteria with Standards Mapping.

Domain	Evaluation Criteria	NIST CSF	ISO 27001
D1: Authentication	· Multi-factor authentication (MFA) · Single sign-on (SSO) support · Biometric authentication · Adaptive authentication · Password policy enforcement	PR.AC-1, PR.AC-6, PR.AC-7	A.9.2, A.9.4
D2: Encryption	· Data at rest encryption (AES-256) · Data in transit encryption (TLS 1.3) · Key management practices · End-to-end encryption · Database-level encryption	PR.DS-1, PR.DS-2, PR.DS-5	A.10.1, A.18.1.5
D3: Access control	· Role-based access control (RBAC) · Attribute-based access control (ABAC) · Principle of least privilege · Segregation of duties · Access review automation	PR.AC-3, PR.AC-4, PR.AC-5	A.9.1, A.9.2
D4: Vulnerability management	· Regular security patching · Vulnerability scanning frequency · Penetration testing · Security incident response · Threat intelligence integration	ID.RA, DE.CM, RS.MI	A.12.6, A.16.1
D5: Compliance	· SOC 2 Type II certification · ISO 27001 certification · HIPAA compliance · GDPR compliance · Industry-specific certifications	ID.GV, PR.IP	A.18.1, A.18.2

*Note:* NIST CSF subcategory identifiers follow version 1.1 notation (e.g., PR.AC, PR.DS), which remains widely used in practitioner guidance and retains a one-to-one conceptual correspondence with CSF 2.0 categories. CSF 2.0 reorganized subcategories under six functions (adding Govern); the mappings above correspond to the 2.0 equivalents as follows: PR.AC $\rightarrow$ PR.AA (Identity Management, Authentication, and Access Control), PR.DS $\rightarrow$ PR.DS (Data Security), ID.RA $\rightarrow$ ID.RA (Risk Assessment), DE.CM $\rightarrow$ DE.CM (Continuous Monitoring), RS.MI $\rightarrow$ RS.MI (Mitigation), ID.GV $\rightarrow$ GV.OC/GV.RM (Organizational Context/Risk Management Strategy), PR.IP $\rightarrow$ PR.PS (Platform Security). We retain version 1.1 notation because it remains the dominant convention in industry compliance documentation referenced by the ERP vendors evaluated. ISO 27001 Annex A control references follow the 2013 numbering convention for backward compatibility; the 2022 edition reorganized these controls under four thematic categories (Organizational, People, Physical, Technological)

### Scoring methodology

Each criterion is evaluated using a 5-point maturity scale aligned with the NIST Cybersecurity Framework Implementation Tiers and the Capability Maturity Model Integration (CMMI) framework (Table 3):

**Table 3:** Security Maturity Scoring Scale.

Score	Maturity level	Description
1	Initial/Ad-hoc	Security practices are undocumented; basic or no implementation of the criterion.
2	Developing	Partial implementation; security measures are reactive rather than proactive.
3	Defined	Documented security processes; consistent implementation across the platform.
4	Managed	Quantitative security metrics; continuous monitoring and improvement processes.
5	Optimizing	Advanced security capabilities; proactive threat prevention; industry-leading practices.

To ensure scoring reproducibility, Table 4 provides criterion-level rubrics for each domain. These rubrics were developed iteratively with input from expert interview participants ($n=12$) and refined through pilot scoring exercises in which three independent assessors applied the rubrics to a subset of three ERP systems, achieving inter-rater reliability of Cohen’s $\kappa =0.82$.

**Table 4:** Criterion-level scoring rubrics by SMAF domain.

Domain	Score	Observable evidence required
D1: Auth	1	Password-only authentication; no MFA option available
	2	Optional MFA available but not enforced by default; basic SSO
	3	MFA enforced for admin accounts; SSO with SAML/OAuth; documented password policy
	4	MFA enforced for all users; SSO with conditional access policies; adaptive/risk-based authentication documented
	5	All of Level 4 plus biometric options, hardware token support, and continuous session validation
D2: Encrypt	1	No documented encryption at rest; TLS version unspecified
	2	TLS 1.2 for transit; encryption at rest available but not verified as AES-256
	3	AES-256 at rest confirmed; TLS 1.2 enforced; vendor-managed keys
	4	AES-256 at rest; TLS 1.2/1.3; customer-managed key options; database-level encryption documented
	5	All of Level 4 plus HSM key storage, end-to-end encryption options, and TLS 1.3 default
D3: Access	1	Basic user/admin role distinction only; no documented RBAC
	2	Configurable roles available; no ABAC; segregation of duties not enforced
	3	RBAC with granular permissions; least-privilege documented; manual access review process
	4	RBAC + ABAC; automated segregation of duties enforcement; scheduled access review workflows
	5	All of Level 4 plus just-in-time access provisioning, micro-segmentation, and API-level access controls
D4: Vuln	1	No documented patch schedule; no vulnerability scanning; no incident response plan
	2	Ad-hoc patching; basic vulnerability notifications; reactive incident response
	3	Documented patch schedule; periodic vulnerability scanning; written incident response procedures
	4	Automated patching; continuous vulnerability scanning; threat intelligence integration; tested incident response. D4 also incorporates disaster recovery (DR) preparedness: RPO/RTO targets, DR plan documentation, and regular DR testing contribute to the domain score.
	5	All of Level 4 plus bug bounty programme, red team exercises, and predictive threat analytics
D5: Comply	1	No third-party security certifications
	2	Self-reported compliance claims without independent audit
	3	SOC 2 Type I or single certification; basic GDPR/HIPAA documentation
	4	SOC 2 Type II; ISO 27001 certified; multiple regulatory compliance frameworks documented
	5	All of Level 4 plus PCI DSS, HIPAA, region-specific certifications, and publicly available audit reports

To establish domain weightings, we conducted a survey of cybersecurity professionals ($n=47$) with expertise in ERP security. Participants were recruited through professional networks including ISACA, (ISC)^2^, and LinkedIn cybersecurity groups during January–March 2024. The survey employed Analytical Hierarchy Process (AHP) pairwise comparisons to derive relative importance weights.

#### Survey participant demographics

Table 5 summarizes the demographic and professional profile of the expert survey respondents.Data for this study were collected through an online expert survey using a structured questionnaire distributed via email to cybersecurity professionals and ERP practitioners with a minimum of five years of relevant industry experience, conducted between January and March 2024. Participants were further selected based on direct experience with ERP system implementation or security assessment and current employment in information security roles. The recruitment yielded a response rate of 58.8% (47 completed out of 80 invited).

**Table 5:** Expert survey participant demographics ($n=47$).

Characteristic	Category	Count	Percentage (%)
Experience (years)	5–10	14	29.8
	11–15	19	40.4
	>15	14	29.8
Industry Sector	Technology / IT Services	18	38.3
	Financial Services	11	23.4
	Manufacturing	9	19.1
	Government / Defence	9	19.1
Professional Certification	CISSP	21	44.7
	CISA / CISM	15	31.9
	CEH / OSCP	7	14.9
	Other	4	8.5
Geographic Region	South Asia (incl. Pakistan)	19	40.4
	Middle East	12	25.5
	North America / Europe	16	34.0

The AHP consistency ratio for all individual responses was verified to be below the 0.10 threshold, ensuring acceptable consistency in pairwise comparisons^17^. Table 6 presents the aggregated domain weights.

*Geographic weight sensitivity:* Given that 40.4% of respondents are from South Asia, we examined whether domain weights differed by region. South Asian respondents assigned marginally higher weight to D1 (Authentication: 0.27 vs. 0.24 for non-South Asian respondents) and lower weight to D4 (Vulnerability Management: 0.16 vs. 0.19). However, Kruskal–Wallis tests across the three geographic groups yielded no statistically significant differences for any domain ($p > 0.10$ for all), and all regional subgroups produced the same domain rank ordering (D1 > D3 > D2 > D4 > D5). The aggregated weights are therefore treated as broadly applicable, though future replication with larger region-stratified samples is warranted (Section “Future research directions”).

**Table 6:** Expert-Derived Domain Weights (AHP Analysis).

Security domain	Weight ($w_i$)	Std. Dev.	Consistency ratio
D1: Authentication	0.25	0.04	0.03
D2: Encryption	0.22	0.05	0.04
D3: Access Control	0.23	0.03	0.02
D4: Vulnerability Management	0.18	0.06	0.05
D5: Compliance	0.12	0.04	0.03
Total	1.00	–	< 0.10

The overall Security Maturity Score (SMS) is calculated using the weighted average formula:

1 $$\begin{aligned} \text {SMS} = \sum _{i=1}^{5} w_i \times S_i \end{aligned}$$

where $w_i$ represents the weight for domain *i* and $S_i$ represents the score for domain *i*.

### Data collection

Security assessments were conducted through multiple data sources to ensure validity through triangulation:

1. *Vendor Documentation Analysis*: Official security whitepapers, compliance certifications, and technical specifications (primary source), collected during June–August 2024.
2. *Third-Party Security Assessments*: SOC 2 reports, penetration testing summaries, and independent security audits where publicly available.
3. *Expert Interviews*: Semi-structured interviews with implementation consultants ($n=12$) specializing in ERP deployments across Pakistan, the Middle East, and North America.
4. *User Feedback Analysis*: Systematic review of security-related feedback from G2, Gartner Peer Insights, and TrustRadius ($n=847$ reviews analyzed), conducted as detailed in Section Data collection.

#### Triangulation protocol

Final maturity scores were determined through a structured triangulation protocol. Vendor documentation served as the primary evidence source and established the baseline score for each criterion. This baseline was then corroborated against third-party assessments (SOC 2 reports, independent audits) where available. Expert interview data was used to adjust scores in cases where implementation realities diverged from vendor claims—for example, where consultants consistently reported that a vendor’s documented feature was not functional or not enabled in typical deployments. User feedback served as a final validation layer: where user reviews systematically contradicted vendor documentation on a specific capability (e.g., multiple reviews reporting MFA failures despite vendor claims of full MFA support), the score was adjusted downward. In cases of conflict between sources, third-party audit evidence was given precedence over vendor documentation, and expert consensus over individual user reports. All score adjustments and their justifications were documented in the scoring matrices (available from the corresponding author upon request).

#### User feedback analysis methodology

User reviews were collected from three enterprise software review platforms (G2, Gartner Peer Insights, and TrustRadius) during the period January 2022 to June 2025. The initial review corpus comprised 3,412 reviews across the eleven ERP systems. Reviews were filtered using a keyword-based approach, retaining only those containing security-related terms including: “security,” “authentication,” “encryption,” “MFA,” “access control,” “vulnerability,” “breach,” “compliance,” “SOC,” “ISO 27001,” “GDPR,” “data protection,” and “zero-trust.” This keyword filtering yielded 847 security-relevant reviews.

The filtered reviews were analyzed using a structured thematic analysis approach^18^. Two researchers independently coded a random subset of 100 reviews to establish inter-coder reliability (Cohen’s $\kappa = 0.78$). Reviews were categorized by (a) SMAF domain (D1–D5), (b) sentiment (positive, neutral, negative), and (c) specific security capability referenced. The resulting thematic categories were used to corroborate and supplement vendor documentation findings, ensuring that user-reported experiences informed the final maturity scores.

### ERP systems under evaluation

Eleven ERP systems were selected based on market presence, deployment diversity (cloud, on-premise, hybrid), and target market segment. The selection includes full-suite ERP platforms (Oracle NetSuite, SAP Business One, Microsoft Dynamics 365), manufacturing-focused systems (Plex Systems, Epicor Prophet 21, SYSPRO), and inventory/operations management systems (inFlow, Fishbowl) that are widely adopted by SMEs as ERP alternatives. Custom Software Solutions is included as a reference category representing bespoke implementations but is excluded from all aggregate statistical analyses due to its implementation-dependent security posture, yielding ten systems in the scored evaluation:

1. Custom Software Solutions (reference category; unscored)
2. inFlow Inventory
3. SAP Business One Professional
4. SYSPRO
5. QT9 ERP
6. Epicor Prophet 21
7. Oracle NetSuite OneWorld
8. Acumatica
9. Microsoft Dynamics 365 Business Central
10. Fishbowl Inventory
11. Plex Systems

## Security evaluation results

This section presents the comprehensive security evaluation results for the eleven ERP systems using the SMAF framework.

### Domain-level security assessment

Table 7 presents the detailed security scores across all five evaluation domains for the ten scored systems.

**Table 7:** Detailed Security Domain Scores by ERP System.

ERP System	D1	D2	D3	D4	D5	SMS
	Auth	Encrypt	Access	Vuln	Comply	(Wtd)
Oracle NetSuite OneWorld	5	5	5	4	5	**4.82**
SAP Business One Pro	5	5	4	4	5	**4.59**
Microsoft Dynamics 365	5	4	5	4	4	**4.48**
Plex Systems	4	5	4	4	4	***4.22***
Acumatica	4	4	4	4	4	***4.00***
Epicor Prophet 21	3	4	4	3	4	*3.57*
SYSPRO	4	4	3	3	3	*3.47*
QT9 ERP	3	3	3	3	4	*3.12*
Fishbowl Inventory	3	3	3	2	3	2.82
inFlow Inventory	2	3	2	2	3	2.34

*Note:* Scores range from 1 (Initial) to 5 (Optimizing). SMS computed via Eq. (1) with weights from Table 6. Custom Software Solutions, representing bespoke implementations with implementation-dependent security postures, is excluded from all scored evaluations and aggregate statistical analyses ($n=10$ scored systems)

To further clarify the distinction between enterprise-grade and SME-targeted solutions, Table 8 presents a statistical comparison by market segment.

**Table 8:** Security Maturity Score Comparison by Market Segment.

Segment	Systems	Mean SMS ($\mu$)	Std. Dev. ($\sigma$)	Min	Max
Enterprise-grade	Oracle, SAP, MS Dynamics	4.63	0.17	4.48	4.82
Mid-market	Plex, Acumatica, Epicor, SYSPRO	3.82	0.35	3.47	4.22
SME-targeted	QT9, Fishbowl, inFlow	2.76	0.39	2.34	3.12

Exact permutation test (one-tailed): Enterprise vs. SME, $p = 0.05$; Enterprise vs. Mid-market, $p = 0.029$. For the Enterprise vs. SME comparison, $p = 0.05$ represents the minimum achievable *p*-value given the sample configuration ($$\left( {\begin{array}{c}6\\ 3\end{array}}\right) = 20$$ permutations). Small per-segment sizes ($n=3$–4) limit statistical power; segment differences should be interpreted primarily as descriptive patterns supported by consistent domain-level score disparities

### Comparative security analysis visualization

**Fig. 5:**
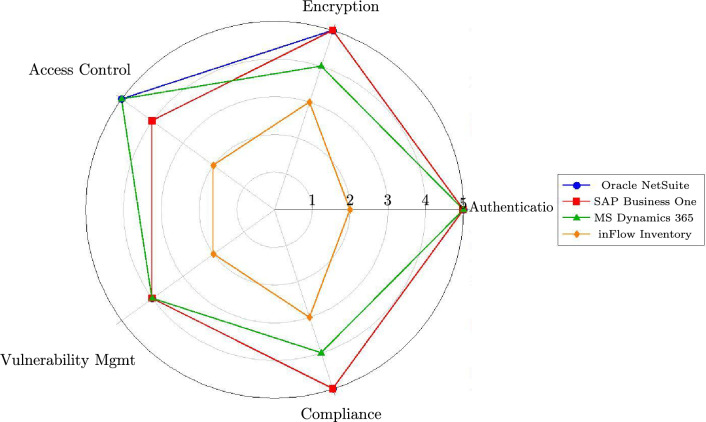
Radar chart comparison of security profiles (selected systems).

Figure 5 presents a radar chart visualization enabling comparison of security profiles across the evaluated systems.

### Detailed compliance metrics analysis

Table 9 presents expanded compliance metrics including specific certification details and encryption standards. Disaster recovery (DR) metrics (RPO/RTO) contribute to D4 (Vulnerability Management) scoring: systems with documented, tested DR plans and aggressive recovery targets (RPO $\le$ 4hr, RTO $\le$ 8hr) receive higher D4 scores, reflecting their readiness to respond to and recover from security incidents.

**Table 9:** Detailed Compliance and Technical Security Specifications.

ERP System	Authentication	Encryption	Certifications	ZTA	DR/BC
Oracle NetSuite OneWorld	MFA, SSO (SAML 2.0), Adaptive Auth^a^	AES-256 (rest), TLS 1.3, HSM	SOC 1/2 Type II, ISO 27001, HIPAA, PCI DSS	Native, micro-seg	RPO: 1hr, RTO: 4hr
SAP Business One Pro	MFA, SSO, Biometric	AES-256, TLS 1.2/1.3, SAP DC	SOC 2 Type II, ISO 27001, HIPAA, GDPR	Partial (BTP)	RPO: 4hr, RTO: 8hr
Microsoft Dynamics 365	MFA (Entra ID), SSO, Conditional^a^	AES-256, TLS 1.2+, Azure KV	SOC 1/2, ISO 27001/27018, HIPAA	Native (Azure ZT)	RPO: 1hr, RTO: 4hr
Plex Systems	MFA, SSO (SAML)	AES-256, TLS 1.2	SOC 2 Type II, ISO 27001	Partial	RPO: 4hr, RTO: 8hr
Acumatica	MFA, SSO (OAuth)	AES-256, TLS 1.2	SOC 1/2 Type II, HIPAA	Limited	RPO: 4hr, RTO: 12hr
Epicor Prophet 21	MFA (opt), Basic SSO	AES-256 (rest)	SOC 2 Type I^b^, GDPR DPA	Not available	RPO: 8hr, RTO: 24hr
SYSPRO	MFA, SSO (LDAP/SAML)	AES-256, TLS 1.2	SOC 2 Type II	Not available	RPO: 4hr, RTO: 12hr
QT9 ERP	Basic MFA	AES-256 (rest)	SOC 2 Type I^b^, FDA 21 CFR Part 11	Not available	RPO: 12hr, RTO: 24hr
Fishbowl Inventory	Basic Auth, Limited MFA	AES-128/256	SOC 2 Type I	Not available	Limited
inFlow Inventory	Password, Opt MFA	AES-256 (cloud), TLS 1.2	SOC 2 Type I	Not available	Basic

*Abbreviations:* MFA = Multi-Factor Authentication, SSO = Single Sign-On, HSM = Hardware Security Module, ZTA = Zero-Trust Architecture, DR = Disaster Recovery, BC = Business Continuity, RPO = Recovery Point Objective, RTO = Recovery Time Objective. DR/BC metrics inform D4 scoring (see Table 4).

^a^Includes device biometric factors (fingerprint, facial recognition) via platform identity provider.

^b^D5 score reflects additional compliance documentation beyond primary certification; see Section “3.4”.

### NIST CSF alignment analysis

**Fig. 6:**
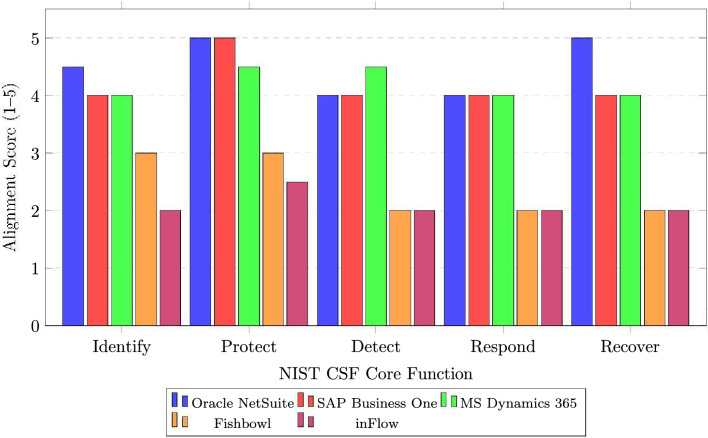
NIST cybersecurity framework alignment by ERP system. *Note:* The five original CSF functions are shown; the Govern (GV) function introduced in CSF 2.0 is addressed through cross-cutting governance criteria in D5 (Compliance).

Figure 6 presents the alignment of evaluated ERP systems with NIST Cybersecurity Framework core functions.

### Emerging technology integration assessment

Table 10 evaluates the current and planned integration capabilities for emerging security technologies across the evaluated systems.

**Table 10:** Emerging Security Technology Integration Status.

ERP System	ZTA	Federated	Blockchain	Digital
		Learning	Audit	Twins
Oracle NetSuite OneWorld	**Implemented**	***Planned***	***Pilot***	***Planned***
SAP Business One Pro	***Partial***	***Planned***	**Available**	**Implemented**
Microsoft Dynamics 365	**Implemented**	***Preview***	***Pilot***	**Implemented**
Plex Systems	***Partial***	*None*	*None*	***Planned***
Acumatica	***Limited***	*None*	*None*	*None*
Epicor Prophet 21	*None*	*None*	*None*	***Planned***
SYSPRO	*None*	*None*	*None*	***Planned***
QT9 ERP	*None*	*None*	*None*	*None*
Fishbowl Inventory	*None*	*None*	*None*	*None*
inFlow Inventory	*None*	*None*	*None*	*None*

*Legend:* **Implemented** = Production; ***Partial/Planned*** = Development; *None* = Not available

## Real-world case studies of ERP security failures

This section examines notable ERP-related security incidents, analyzing vulnerabilities exploited and lessons learned through the lens of the SMAF framework. Beyond retrospective analysis of well-documented international incidents, we include two publicly documented cybersecurity incidents from Pakistan, analyzed using evidence from published institutional statements, investigative journalism, and regulatory filings to provide actionable guidance aligned with the SMAF domains.

### Target corporation breach (2013)

The Target breach represents a seminal case in ERP-adjacent security failures. Attackers exploited third-party vendor credentials (Fazio Mechanical Services, an HVAC contractor) to gain network access, subsequently pivoting to Target’s point-of-sale systems integrated with ERP infrastructure^19^.

**SMAF Domain Analysis:**

- *D1 (Authentication)*: Failure—vendor access lacked MFA requirements.
- *D3 (Access Control)*: Failure—inadequate network segmentation between vendor systems and critical infrastructure.
- *D4 (Vulnerability Management)*: Failure—alerts from security tools were not acted upon.

**Remediation Measures:** Target invested $100M+ in security enhancements including chip-and-PIN implementation, network segmentation, and enhanced vendor security requirements.

**Estimated Pre-Breach SMS:** Applying SMAF retrospectively based on publicly documented security posture at the time of breach yields estimated scores of D1: 2 (no vendor MFA), D2: 4 (encryption present), D3: 2 (inadequate segmentation), D4: 2 (alerts ignored), D5: 4 (PCI DSS compliant), producing an estimated SMS of $\approx$2.68—comparable to current SME-tier solutions and underscoring how targeted weaknesses in authentication and access control can undermine otherwise adequate security investments.

### Equifax Breach (2017)

The Equifax breach exposed 147 million consumer records due to an unpatched Apache Struts vulnerability (CVE-2017-5638) in a web-facing application connected to ERP systems^20^.

**SMAF Domain Analysis:**

- *D2 (Encryption)*: Partial failure—data at rest encryption was inconsistent.
- *D4 (Vulnerability Management)*: Critical failure—patch available 2 months before breach but not applied.
- *D5 (Compliance)*: Failure—inadequate security monitoring despite regulatory requirements.

**Estimated Pre-Breach SMS:** Retrospective SMAF application yields D1: 3, D2: 2 (inconsistent encryption), D3: 3, D4: 1 (critical patch failure), D5: 3, producing SMS $\approx$2.42. The catastrophic D4 score reflects a systemic vulnerability management failure that no other domain strength could compensate for, illustrating the importance of the SMAF’s domain-level visibility over aggregate scores alone.

### SolarWinds supply chain attack (2020)

The SolarWinds attack compromised the Orion software platform, affecting approximately 18,000 organizations including government agencies with ERP integrations^21^.

**SMAF Domain Analysis:**

- *D3 (Access Control)*: Failure—excessive privileges granted to monitoring software.
- *D4 (Vulnerability Management)*: Systemic failure—supply chain integrity not verified.

**Fig. 7:**
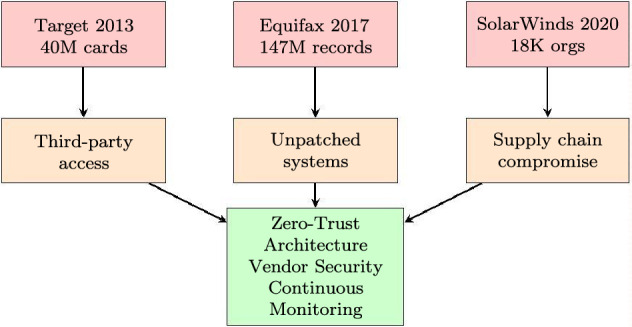
ERP security breach analysis: attack vectors and lessons learned.

**Implications for ERP Security:** This attack highlighted the critical importance of supply chain security for ERP systems that integrate with numerous third-party tools and services (Fig. 7).

### ERP-adjacent security failures in Pakistan: evidence from published incidents

To ground the SMAF framework in the Pakistani domestic context, this section analyzes two publicly documented cybersecurity incidents involving enterprise financial management systems in Pakistan. Both cases are examined through the SMAF evaluation domains using evidence from published institutional statements, investigative journalism, and regulatory filings. These cases demonstrate the practical relevance of the SMAF framework for assessing security postures in developing economy contexts.

*Note on source material:* The Pakistani case studies rely on published media reports (The Express Tribune, Dawn, HackRead), government advisory databases (CISA), and ransomware tracking platforms (Ransomware.live) rather than direct forensic investigation. While these sources are not peer-reviewed, they represent the best available public evidence for these incidents, consistent with the case study methodology employed by Krebs ^19^ for the Target breach analysis. SMAF scores assigned to these incidents are best estimates and should be interpreted accordingly.

#### Case study A: federal board of revenue (FBR) cyber attack (August 2021)

**Background:** The Federal Board of Revenue (FBR), Pakistan’s apex federal revenue collection agency operating the country’s largest data centre and tax/revenue ERP backbone through the Pakistan Revenue Automation Limited (PRAL), was subjected to a major cyber attack on 14 August 2021^22,23^. Attackers exploited vulnerabilities in Microsoft Hyper-V software, compromising approximately 360 virtual machines—nearly half the total—and bringing all FBR websites, the IRIS e-filing system, and the WeBOC customs clearance system offline for over 72 hours^22^. Network access to the compromised systems was subsequently offered for sale on a Russian cybercrime forum for $26,000^23^. The attack occurred despite advance warnings from Pakistan’s intelligence services about an imminent cyber threat^24^ (Table 11).

**SMAF Domain Analysis:**

**Table 11:** SMAF Assessment: FBR Data Centre Attack (August 2021).

Domain	Score	Evidence and Justification
D1: Authentication	2	Spearphishing emails with spoofed government addresses enabled initial access; administrator credentials were compromised, suggesting inadequate MFA enforcement for privileged accounts^25^.
D2: Encryption	1	The use of pirated and outdated Microsoft Hyper-V software indicated systemic neglect of infrastructure security, making it unlikely that encryption was properly configured or maintained. The World Bank had flagged “end-of-life equipment” since 2019, and legacy unpatched software was in use^22^. No evidence of data-at-rest encryption or key management practices was documented.
D3: Access Control	2	Once the Hyper-V layer was compromised, attackers accessed approximately 360 of 720 virtual machines, indicating inadequate network segmentation and excessive lateral movement capabilities^22^.
D4: Vulnerability Mgmt	1	Intelligence services had warned FBR days before the attack; warnings were ignored. The World Bank’s 2019 and 2021 reports documented obsolete ICT equipment and delayed procurement of security upgrades. An $80M loan allocation for IT infrastructure was largely unspent on its intended purpose^22,24^.
D5: Compliance	1	No known third-party security certifications. No disaster recovery plan was activated—services required manual recreation of virtual environments. No cybersecurity audit had been conducted despite the 2019 breach^24^.
Weighted SMS: $$\mathbf {(0.25 \times 2) + (0.22 \times 1) + (0.23 \times 2) + (0.18 \times 1) + (0.12 \times 1) = 1.48}$$		

**Key Findings:** The FBR case yields an estimated SMS of 1.48, representing the lowest score in our study and illustrating catastrophic security posture failure across all five SMAF domains. The case demonstrates three critical patterns relevant to ERP security in developing economies: (1) intelligence-derived threat warnings were ignored due to organizational inertia (D4); (2) earmarked modernization funding ($80M World Bank loan) was not directed toward its intended IT security purpose (D2, D5); and (3) the absence of business continuity planning meant that a single successful attack disabled the entire national tax collection infrastructure for over 72 hours (D5).

#### Case study B: NIFT ransomware attack (June 2023)

**Background:** The National Institutional Facilitation Technologies (NIFT), Pakistan’s sole automated cheque clearing house processing 150,000–160,000 cheques daily for 67.5 million bank accounts holding approximately PKR 23 trillion in deposits, was attacked by the ALPHV/BlackCat ransomware group on 16 June 2023^26^. Both data centres (Islamabad and Karachi) were shut down, forcing nationwide manual cheque clearing for over seven days. The ALPHV group claimed responsibility and listed NIFT as a victim on their dark web leak site, with the estimated attack date of 22 June 2023 confirmed by ransomware tracking platforms^27^ (Table 12).

**SMAF Domain Analysis:**

**Table 12:** SMAF Assessment: NIFT Ransomware Attack (June 2023).

Domain	Score	Evidence and Justification
D1: Authentication	2	Unauthorized access was achieved, suggesting authentication weaknesses. ALPHV/BlackCat affiliates are documented to use credential theft, MFA bypass via Evilginx2, and social engineering for initial access^28^.
D2: Encryption	2	Sensitive data including cheque scans and customer records was accessible post-breach. Despite NIFT claiming “no significant compromise,” IT experts expressed concerns that 67.5 million customer records were put at risk^26^.
D3: Access Control	2	Once breached, extensive data was accessible, indicating inadequate micro-segmentation between operational systems and data repositories. Both Islamabad and Karachi data centres were affected simultaneously^26^.
D4: Vulnerability Mgmt	1	The attack was not detected and contained rapidly enough to prevent operational disruption. NIFT’s disaster recovery (DR) system, designed to restore services within hours, failed to function as intended—recovery took over seven days^26^.
D5: Compliance	2	State Bank of Pakistan (SBP) oversight proved insufficient; no prior compliance audit had identified the vulnerabilities exploited. SBP subsequently requested NIFT to submit future safety measures^26^.
Weighted SMS: $\mathbf {(0.25 \times 2) + (0.22 \times 2) + (0.23 \times 2) + (0.18 \times 1) + (0.12 \times 2) = 1.82}$		

*Key Findings:* The NIFT case yields an estimated SMS of 1.82. Despite NIFT being a systemically important financial institution processing the entire nation’s cheque clearing, its security posture was comparable to the lowest-tier SME ERP solutions evaluated in Section “Security evaluation results”. The case reveals a critical finding: (1) disaster recovery systems that are not regularly tested may provide false confidence (D4, D5); (2) regulatory oversight (SBP supervision) alone is insufficient without mandated security assessments aligned to international standards (D5); and (3) the absence of network segmentation between data centres meant that a single attack vector disabled both primary and backup facilities simultaneously (D3).

#### Comparative analysis and implications for Pakistani context

Table 13 compares the SMAF scores of the two Pakistani incidents with the international breach cases and the SMAF evaluation results.

**Table 13:** SMAF Comparison: Pakistani Incidents vs. International Breaches and ERP Evaluation.

Case	D1	D2	D3	D4	D5	SMS
FBR 2021 (Pakistan)	2	1	2	1	1	**1.48**
NIFT 2023 (Pakistan)	2	2	2	1	2	**1.82**
Target 2013 (USA)	2	4	2	2	4	***2.68***
Equifax 2017 (USA)	3	2	3	1	3	***2.42***
*Lowest ERP evaluated (inFlow)*	*2*	*3*	*2*	*2*	*3*	*2.34*

Three findings emerge from the Pakistani cases. First, the SMAF scores for both FBR and NIFT fall *below* the lowest vendor-evaluated ERP system (inFlow, SMS: 2.34), confirming that organizational implementation failures can produce security postures worse than even the most basic commercial ERP offerings. Second, D4 (Vulnerability Management) is the weakest domain in both Pakistani cases (score: 1), consistent with the broader finding from the ERP evaluation that vulnerability management remains the primary differentiator between enterprise and lower-tier solutions. Third, D5 (Compliance) failures in the Pakistani context are distinct from the international cases: whereas Target and Equifax held formal certifications (PCI DSS, regulatory compliance) that proved insufficient against specific attack vectors, FBR and NIFT lacked any independent security certification or audit framework, suggesting that in developing economies, the absence of basic compliance infrastructure—rather than compliance gaps—is the fundamental challenge.

These findings directly inform the SME-specific recommendations in Section “SME-specific security recommendations”, particularly the emphasis on mandatory vulnerability management processes and independent security certification as minimum viable security measures.

## Discussion

### Key findings

The application of the SMAF framework reveals significant disparities in security maturity across ERP platforms:

1. *Enterprise vs. SME Solutions*: Enterprise-targeted solutions (Oracle NetSuite, SAP, Microsoft Dynamics) demonstrate consistently higher security maturity (mean SMS: $\mu =4.63$, $\sigma =0.17$) compared to mid-market solutions ($\mu =3.82$, $\sigma =0.35$) and SME-focused solutions ($\mu =2.76$, $\sigma =0.39$). The 68% gap between enterprise and SME segments (Table 8) presents significant risk implications, though we note that small per-segment sample sizes ($n=3$–4) limit formal statistical inference; these differences should be interpreted primarily as descriptive patterns supported by consistent domain-level score disparities. Notably, this disparity is most pronounced in domain D4 (Vulnerability Management), where enterprise solutions average 4.0 compared to 2.3 for SME solutions, and least pronounced in D5 (Compliance), where even lower-tier solutions achieve Type I certifications. This suggests that compliance certifications have become commoditized, while proactive vulnerability management remains a key differentiator.
2. *Cloud vs. On-Premise*: Cloud-native solutions exhibit superior security postures in D1 (Authentication) and D5 (Compliance) domains, attributed to centralized security management and vendor-maintained infrastructure. However, on-premise solutions offer advantages in data sovereignty and customization flexibility, which may be important for organizations in jurisdictions with strict data localization requirements.
3. *Emerging Technology Adoption*: ZTA adoption correlates strongly with overall security maturity (Spearman $r_s=0.87$, $p<0.01$, $n=10$). However, this correlation should be interpreted cautiously: ZTA principles map directly to D1, D3, and D4, which together account for 66% of the weighted SMS. Consequently, the correlation partially reflects the mechanical relationship between ZTA-aligned capabilities and the SMAF scoring structure rather than an independent validation of ZTA’s security value. Nevertheless, the correlation confirms that the SMAF appropriately captures the security benefits associated with zero-trust adoption. Federated learning and blockchain integration remain nascent, with only tier-1 vendors demonstrating production implementations.
4. *Compliance as Differentiator*: SOC 2 Type II certification serves as a meaningful quality indicator, with certified systems scoring 1.3 points higher on average than Type I or uncertified alternatives.
5. *Implementation and Institutional Gaps*: The Pakistani case studies (Section “ERP-adjacent security failures in Pakistan: evidence from published incidents”) demonstrate that actual security postures of national-level financial institutions can fall significantly below even the lowest-tier commercial ERP solutions when basic security practices are absent. Both FBR (SMS: 1.48) and NIFT (SMS: 1.82) scored below inFlow Inventory (SMS: 2.34), underscoring that vendor capability is necessary but insufficient—organizational commitment to security implementation is the critical determinant.

### Framework validation

The SMAF framework demonstrates strong internal consistency (Cronbach’s $\alpha =0.89$) and inter-rater reliability (Cohen’s $\kappa =0.82$) based on independent assessments by three security professionals. Expert survey participants rated the framework’s practical utility at 4.2/5.0 for organizational decision-making.

### Limitations

This study acknowledges several limitations:

- *Vendor Data Dependence*: Security assessments rely partially on vendor-provided documentation, which may present optimistic characterizations. This limitation is most acute for lower-tier systems (Fishbowl, inFlow, QT9) where independent third-party assessments are limited or unavailable. Multi-source triangulation through user reviews and expert interviews mitigates but does not eliminate this bias (see Section “3.4” for the triangulation protocol). Future work should incorporate independent penetration testing results where available.
- *Temporal Validity*: ERP security features evolve rapidly; assessments reflect capabilities as of Q3 2024.
- *Implementation Variability*: Actual security postures depend significantly on customer implementation choices beyond vendor-provided defaults, as demonstrated by the Pakistani case studies.
- *Geographic Scope*: Compliance certifications assessed are primarily US and EU-centric; regional variations exist. Future work should incorporate region-specific standards.
- *Survey Sample*: While the expert survey ($n=47$) exceeds typical AHP study samples and achieves acceptable consistency ratios, expanding the participant pool to include a broader geographic and sectoral representation would further strengthen the weight derivation.
- *Ordinal Scale Averaging*: The SMAF employs a 5-point ordinal maturity scale; computing weighted averages of ordinal data assumes equidistant intervals between maturity levels. While this assumption is standard in CMMI-based assessments, users should interpret SMS differences of less than 0.5 points with appropriate caution.
- *System Selection Scope*: The eleven systems evaluated include both full-suite ERP platforms and inventory/manufacturing management systems that serve as ERP alternatives for SMEs. While all systems manage enterprise data and face comparable security challenges, readers should note that inFlow and Fishbowl are primarily inventory management tools and may not be directly comparable to full-suite ERP systems across all functional dimensions.
- *Case Study Limitations*: The Pakistani case studies (Section “ERP-adjacent security failures in Pakistan: evidence from published incidents”) rely on published media reports and institutional statements rather than direct forensic investigation. SMAF scores assigned to these incidents represent best estimates based on publicly available evidence and should be interpreted accordingly.

### Implications for practice

Organizations selecting ERP systems should:

1. Prioritize authentication (D1) and access control (D3) capabilities, which received highest expert weightings.
2. Require SOC 2 Type II certification as a minimum compliance threshold.
3. Evaluate ZTA support for future-proofing security investments.
4. Consider total cost of security, including implementation resources beyond licensing fees.
5. Use the SMAF framework for structured pre-procurement security evaluation.

## Recommendations for secure ERP implementation

Based on the SMAF evaluation results and case study analysis, we provide the following evidence-based recommendations organized by implementation phase (Table 14).

**Table 14:** Prioritized Security Recommendations by Implementation Phase.

Phase	Recommendation	Implementation Guidance	Priority
Pre- Implem.	Conduct Risk Assessment	Map organizational data assets to ERP modules; identify regulatory requirements	Critical
	Verify Vendor Certifications	Request SOC 2 Type II reports; validate ISO 27001 certification scope	Critical
	Evaluate ZTA Readiness	Assess vendor’s identity management integration capabilities	High
Implem.	Enforce MFA	Enable multi-factor authentication for all user accounts without exception	Critical
	Implement RBAC	Define roles based on least-privilege principle; document access matrices	Critical
	Configure Encryption	Enable AES-256 encryption at rest; verify TLS 1.2+ for all connections	High
Post- Implem.	Establish Monitoring	Deploy SIEM integration; configure alerting thresholds	High
	Develop Incident Response	Create ERP-specific incident response procedures; conduct tabletop exercises	High
	Schedule Security Reviews	Conduct quarterly access reviews; annual penetration testing	Medium

### SME-specific security recommendations

The Pakistani case studies (Section “ERP-adjacent security failures in Pakistan: evidence from published incidents”) and the ERP evaluation results identified systematic security gaps in developing economy contexts. To address these gaps, we provide targeted recommendations for SMEs and institutions in developing economies, organized by SMAF domain and resource requirements.

**Table 15:** SME-Oriented Security Recommendations by SMAF Domain.

Domain	Recommendation	Cost	Priority
D1: Auth	Enforce MFA for all users; use SSO where supported to reduce password fatigue; eliminate shared credentials	Low	Critical
D2: Encrypt	Verify AES-256 is active at rest; confirm TLS 1.2+ for all connections; ensure licensed and current software is in use	Low	Critical
D3: Access	Conduct access role audit; remove excessive admin privileges; implement network segmentation between critical subsystems	Low	Critical
D4: Vuln Mgmt	Enable automatic vendor patches; act on threat intelligence warnings; establish and regularly test incident response and disaster recovery plans	Low–Med	Critical
D5: Comply	Require vendors to provide SOC 2 Type II reports; conduct or commission independent security audits; document data processing agreements	Low–Med	High

*Cost:* Low = no additional licensing; Med = moderate consulting/tooling investment

**Product Recommendations for SMEs:** Based on the SMAF evaluation results (reflecting Q3 2024 capabilities), SMEs with limited security budgets should consider Acumatica (SMS: 4.00) or QT9 ERP (SMS: 3.12) as cost-effective alternatives to enterprise platforms, provided they implement the security hardening measures in Table 15. For organizations in regulated industries (e.g., healthcare, financial services), the minimum recommended SMS threshold is 4.0, suggesting Plex Systems (SMS: 4.22) or Acumatica as the most appropriate mid-market options. Organizations for which data sovereignty is a primary concern should evaluate SYSPRO (SMS: 3.47) for its on-premise deployment options with appropriate security hardening. Critically, the FBR and NIFT cases demonstrate that even large institutions can exhibit security postures below SME-tier ERP solutions when basic practices (patching, DR testing, independent audits) are neglected—reinforcing that *how* a system is implemented matters as much as *which* system is selected.

## Future research directions

The findings of this study, together with the identified limitations and emerging technology landscape, motivate several specific research directions. Table 16 outlines these directions with explicit linkage to the current findings.

**Table 16:** Future Research Directions in ERP Security.

Research Area	Description and Linkage to Current Findings	Methodological Approach
AI-Driven Threat Detection	Development of machine learning models for real-time anomaly detection in ERP transaction patterns. Addresses D4 gap identified in Pakistani case studies (Section “ERP-adjacent security failures in Pakistan: evidence from published incidents”).	Supervised/ unsupervised ML on ERP log data
Blockchain Audit Integrity	Evaluation of blockchain-based immutable audit trails for ERP compliance verification. Extends Table 10 pilot implementations to production validation.	Prototype development; performance benchmarking
Federated Learning Privacy	Assessment of privacy-preserving analytics frameworks for multi-tenant ERP environments. Addresses D2/D5 enhancement identified in Table 1.	Differential privacy analysis; utility evaluation
Digital Twin Security Simulation	Creation of virtual ERP environments for security testing and incident simulation. Extends D4 capabilities for predictive vulnerability assessment.	Simulation modeling; attack scenario testing
Quantum-Resistant Cryptography	Evaluation of post-quantum cryptographic algorithms for long-term ERP data protection.	Cryptographic protocol analysis
Regional SMAF Validation	Replication of SMAF evaluation with region-specific compliance standards and expanded expert samples ($n > 100$). Addresses survey sample and geographic scope limitations.	Cross-cultural AHP; multi-country expert panels

## Conclusion

This study developed and applied the Security Maturity Assessment Framework (SMAF) to evaluate eleven leading ERP systems across five security domains grounded in NIST CSF 2.0 and ISO/IEC 27001:2022 standards. The framework addresses a critical gap in ERP security literature by providing reproducible, quantifiable security assessments validated through expert surveys ($n=47$), multi-source data triangulation, and systematic user feedback analysis ($n=847$ reviews).

Key findings demonstrate substantial security maturity disparities between enterprise-grade solutions (SMS range: 4.48–4.82), mid-market solutions (SMS range: 3.47–4.22), and SME-targeted alternatives (SMS range: 2.34–3.12). Oracle NetSuite OneWorld, SAP Business One Professional, and Microsoft Dynamics 365 Business Central emerge as security leaders, featuring comprehensive authentication mechanisms, advanced encryption protocols, and extensive compliance certifications. The analysis of two published cybersecurity incidents in Pakistan (FBR 2021, SMS: 1.48; NIFT 2023, SMS: 1.82) further demonstrates that institutional security postures in developing economies can fall below even the lowest-tier commercial ERP solutions when basic security practices are absent, underscoring the need for structured assessment tools.

The analysis of emerging security paradigms reveals that ZTA integration correlates with overall security maturity (Spearman $r_s=0.87$, $p<0.01$), though this correlation partially reflects the mechanical overlap between ZTA-aligned domains and the SMAF weighting structure rather than serving as an independent validation of ZTA’s security value. Federated learning and blockchain implementations remain early-stage across most platforms. Digital twin integration for Industry 5.0 alignment presents significant opportunities for enhanced security monitoring but requires substantial development investment.

Organizations selecting ERP systems should prioritise authentication and access control capabilities, require SOC 2 Type II certification, evaluate vendor roadmaps for emerging technology adoption, and—critically for developing economy contexts—apply the SMAF as a structured assessment tool to identify and remediate institutional-level security gaps. The SMAF framework provides a practical, reproducible methodology for evidence-based ERP security evaluation, enabling informed decision-making aligned with organizational risk tolerance and regulatory requirements.

## Data Availability

The survey data and detailed scoring matrices supporting the findings of this study are available from the corresponding author upon reasonable request, subject to participant confidentiality and privacy requirements.
